# Power Modulations of ECoG Alpha/Beta and Gamma Bands Correlate With Time-Derivative of Force During Hand Grasp

**DOI:** 10.3389/fnins.2020.00100

**Published:** 2020-02-14

**Authors:** Tianxiao Jiang, Giuseppe Pellizzer, Priscella Asman, Dhiego Bastos, Shreyas Bhavsar, Sudhakar Tummala, Sujit Prabhu, Nuri F. Ince

**Affiliations:** ^1^Clinical Neural Engineering Lab, Biomedical Engineering Department, University of Houston, Houston, TX, United States; ^2^Research Service, Minneapolis VA Health Care System, Departments of Neurology and Neuroscience, University of Minnesota, Minnesota, MN, United States; ^3^Department of Neurosurgery, University of Texas MD Anderson Cancer Center, Houston, TX, United States; ^4^Department of Anesthesiology, University of Texas MD Anderson Cancer Center, Houston, TX, United States

**Keywords:** high-density ECoG, hand grasp, ERD, ERS, time-derivative of force

## Abstract

It is well-known that motor cortical oscillatory components are modulated in their amplitude during voluntary and imagined movements. These patterns have been used to develop brain-machine interfaces (BMI) which focused mostly on movement kinematics. In contrast, there have been only a few studies on the relation between brain oscillatory activity and the control of force, in particular, grasping force, which is of primary importance for common daily activities. In this study, we recorded intraoperative high-density electrocorticography (ECoG) from the sensorimotor cortex of four patients while they executed a voluntary isometric hand grasp following verbal instruction. The grasp was held for 2 to 3 s before being instructed to relax. We studied the power modulations of neural oscillations during the whole time-course of the grasp (onset, hold, and offset phases). Phasic event-related desynchronization (ERD) in the low-frequency band (LFB) from 8 to 32 Hz and event-related synchronization (ERS) in the high-frequency band (HFB) from 60 to 200 Hz were observed at grasp onset and offset. However, during the grasp holding period, the magnitude of LFB-ERD and HFB-ERS decreased near or at the baseline level. Overall, LFB-ERD and HFB-ERS show phasic characteristics related to the changes of grasp force (onset/offset) in all four patients. More precisely, the fluctuations of HFB-ERS primarily, and of LFB-ERD to a lesser extent, correlated with the time-course of the first time-derivative of force (yank), rather than with force itself. To the best of our knowledge, this is the first study that establishes such a correlation. These results have fundamental implications for the decoding of grasp in brain oscillatory activity-based neuroprosthetics.

## 1. Introduction

Brain-machine interfaces (BMI) provide a way to establish voluntary control of prosthetic limbs by decoding signals directly from the sensorimotor area of the brain (Yanagisawa et al., 2011, 2012; Hochberg et al., 2012; Collinger et al., 2013; Wang et al., 2013; Wodlinger et al., 2015). Event-related desynchronization (ERD) in low frequency band (LFB, 8–32 Hz) and event-related synchronization (ERS) in high frequency band (HFB, 60–200 Hz) are two well-known forms of frequency modulations associated with movements in human (Pfurtscheller and Lopes da Silva, 1999; Miller et al., 2007, 2010; Tzagarakis et al., 2010; Su and Ojemann, 2013; Jiang et al., 2017, 2018) and non-human primates (Sanes and Donoghue, 1993; Ray et al., 2008; Ince et al., 2010; Chen et al., 2014). ERD and ERS modulations have been used successfully in various BMI applications. However, most BMI studies have focused exclusively on decoding movement kinematics, such as individual finger position and velocity (Kubánek et al., 2009; Miller et al., 2009; Acharya et al., 2010; Hochberg et al., 2012; Nakanishi et al., 2014; Hotson et al., 2016; Branco et al., 2017; Flint et al., 2017). In contrast, movement kinetic factors, such as the control of grasp force, are less often studied even though they are of primary importance for the proper function of neuroprostheses in real-world applications (Pistohl et al., 2012; Chen et al., 2014; Flint et al., 2014).

Cramer et al. found in a functional magnetic resonance imaging (fMRI) study that increasing hand squeezing force correlated significantly with the increase of blood-oxygen-level-dependent (BOLD) signal in the contralateral sensorimotor region (Cramer et al., 2002). However, due to the limited temporal resolution of fMRI, the study could not investigate the different phases of hand grasp in detail. To date, only a handful of studies have attempted to decode grasp forces using brain oscillatory activity recorded from human (Flint et al., 2014) and non-human primates (Shin et al., 2012; Chen et al., 2014). Shin et al. found a strong correlation between the activity of the flexor digitorum profundus finger muscle and the power in the delta (1.4–4 Hz) and gamma (50–90 Hz) subbands (Shin et al., 2012). Chen et al. found that the lateral grasp force correlated mainly with the power in the gamma band from the monkey motor cortex (Chen et al., 2014). Flint et al. decoded the continuous isometric pinch force generated between the thumb and the index or little finger using local motor potentials and high gamma-band power of human sensorimotor electrocorticography (ECoG) (Flint et al., 2014). However, in all of these studies, either the force generated was pulse-like, that is, the task was to generate a grasp force and relax without actually holding the grasp force for a prolonged period of time, or only the onset (squeeze) phase of the grasp was analyzed. For these reasons, the relation between grasp force during the different phases of grasp (onset, hold, offset) and brain oscillatory activity is essentially unknown. In addition, as we will show in the results, time separation between the onset (squeeze), hold (steady force), and offset (relaxation) phases of the grasp is crucial for clarifying the relation between the time-course of grasp force and oscillatory neural activity.

Here we investigated the relation between power modulations in oscillatory brain activity and grasp force during onset, hold, and offset phases. To this end, we obtained intraoperative high-density ECoG recordings over the sensorimotor areas of four patients undergoing awake neurosurgery while they were instructed to perform an isometric grasp task. We observed phasic LFB-ERD and HFB-ERS patterns at the grasp onset and offset, but not during the hold period. In addition, we found that the time-course of HFB-ERS, and to a lesser extent LFB-ERD, correlated strongly with the first-time-derivative of grasp force rather than with the force itself.

## 2. Methods and Materials

### 2.1. Patients

Four patients (2 females and 2 males; ages within 40–65 years) scheduled for resection surgeries requiring a craniotomy in the vicinity of their sensorimotor area were recruited in this study. All four patients were diagnosed with a brain tumor and had been admitted at the University of Texas MD Anderson Cancer Center (Houston, TX). Behavioral task examinations were carried out one day before the surgery to exclude motor deficits for all patients. Specifically, the patients were trained such that they get familiar with the experimental paradigm.

Awake craniotomy with intraoperative ECoG functional mapping was performed in all four cases. The sleep-awake-sleep anesthetic technique was adopted as described by Huncke et al. (1998). All patients received regional scalp block anesthesia. During the ECoG mapping, there were no general anesthetic effects verified on ECoG showing a continuous reactive background with no suppression. During the awake period of the operation, the patient was asked to perform verbal and visual tasks to facilitate the identification of speech and motor areas during cortical stimulation. Motor and sensory cortices were identified with cortical stimulation and/or phase reversals obtained following contralateral upper extremity (median nerve) somatosensory evoked potentials. Simultaneous recordings of electrocorticography did not show epileptiform discharges (cortical irritability) or subclinical electrographic seizures in all four patients. After cortical stimulation, hand grasp tasks were performed.

The study protocol was reviewed and approved by the Institutional Review Boards (IRB) of the MD Anderson Cancer Center and the University of Houston. Informed consent was obtained from all four patients before their participation in the study in accordance with the Declaration of Helsinki. Patient demographics and clinical information, as well as the hand used for the grasps and number of trials analyzed, are provided in Table 1.

**Table 1:** Summary of patients and experiments.

**ID**	**Tumor site**	**Tumor type**	**Hand**	**Trials (onset/offset)**
P1	R superior frontal gyrus	WHO grade II oligodendroglioma	Left	27/26
P2	L frontal motor gyrus	Metastatic adenocarcinoma	Right	31/25
P3	L superior parietal lobule	WHO grade II oligodendroglioma	Right	14/12
P4	R middle frontal gyrus	WHO grade II astrocytoma	Left	39/43

### 2.2. ECoG Recordings

**Figure 1:**
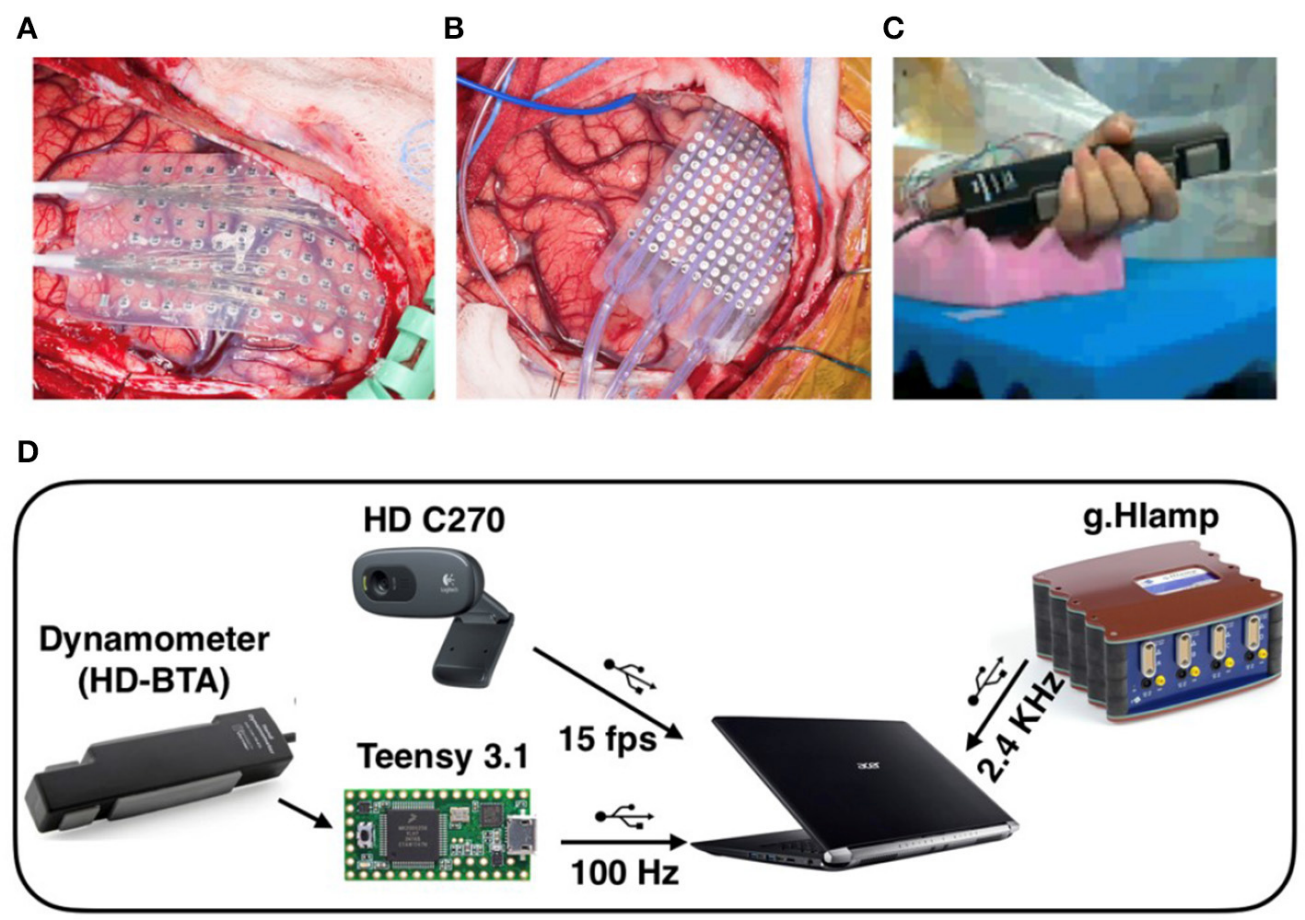
**(A)** High-density ECoG grid used for P1 (16 x 8, 4 mm spacing, AdTech). **(B)** High-density ECoG grid used for P2, P3, and P4 (16 x 12, 3 mm spacing, PMT). **(C)** Patient squeezing the hand dynamometer with forearm and hand supported with convoluted foam during awake surgery. **(D)** Recording system setup used in the operating room. The Teensy microcontroller digitized the dynamometer output and transmitted the reading at 100 Hz over USB to a laptop computer. The ECoG and EMG data were amplified, digitized, and acquired at 2.4 kHz. In addition, an HD video of the active hand was recorded at 15 frames per second. All data streams over USB connections were synchronized in Matlab/Simulink.

High-density ECoG was recorded from four patients intraoperatively during awake craniotomy. A customized 128 channel grid (16 × 8, 1.17 mm contact exposure and 4 mm spacing, platinum, Ad-Tech, Michigan, MI) was used for P1. Customized 192 channel grids (16 × 12, 1 mm contact exposure and 3 mm spacing, platinum, PMT, Chanhassen, MN) were used for P2, P3, and P4. Intraoperative recordings pose challenges in experimental design in terms of both robustness and portability given patient tolerability, limited time and space in the operating room. The portable data acquisition system setup used in this study, shown in Figure 1, was specifically designed for intraoperative neural and behavioral recordings (Jiang et al., 2017).

During the awake surgery, patients were instructed verbally to tightly squeeze a dynamometer (Vernier HD-BTA) with the whole hand contralateral to the electrode grid placement and maintain the grip for 2 to 3 s until instructed to relax. Since the recordings were obtained intraoperatively, it is difficult to provide visual feedback to the subjects. Therefore, no explicit target force was specified, and no force feedback was given during the task. The subjects were trained one day before the surgery for the grip task and asked to maintain their grip force at a steady level. We assumed that such a grip task was more natural and reminiscent of the real-life setting as we grip objects and keep that grip force at a steady level by using our somatosensory feedback rather than visual feedback. The hand and forearm used in the task of each patient were supported with convoluted foam to limit elbow flexion and other unnecessary movements (Figure 1). We also suspended the experiment and asked the patients to completely relax when we observed repetitive bursts of EMG during relaxation. An inter-trial interval of approximately 3 s was maintained between the instruction of hand relaxation and the consecutive hand squeezing instruction. Patients were given a short break of 30–40 s after 10–15 trials to prevent fatigue. Hand movements were monitored using a high-definition webcam (Logitech HD C270). Grasp force was measured with an analog hand dynamometer (Vernier HD-BTA) and digitized using a microcontroller (Teensy 3.1) at 100 Hz and 12 bit A/D resolution. The digitized force was transferred to a nearby laptop over the User Datagram Protocol using an in-house made software (Figure 1) (Jiang et al., 2017). The neural data and forearm bipolar electromyogram (EMG) were recorded with a 256 channel clinical bioamplifier (gHIamp, g.tec medical engineering GmbH, Graz Austria) at 2.4 kHz. All behavioral and neural data were acquired, synchronized and visualized in real-time intraoperatively using Simulink/Matlab and gHIsys block sets (g.tec medical engineering GmbH, Graz Austria).

### 2.3. Preprocessing

**Figure 2:**
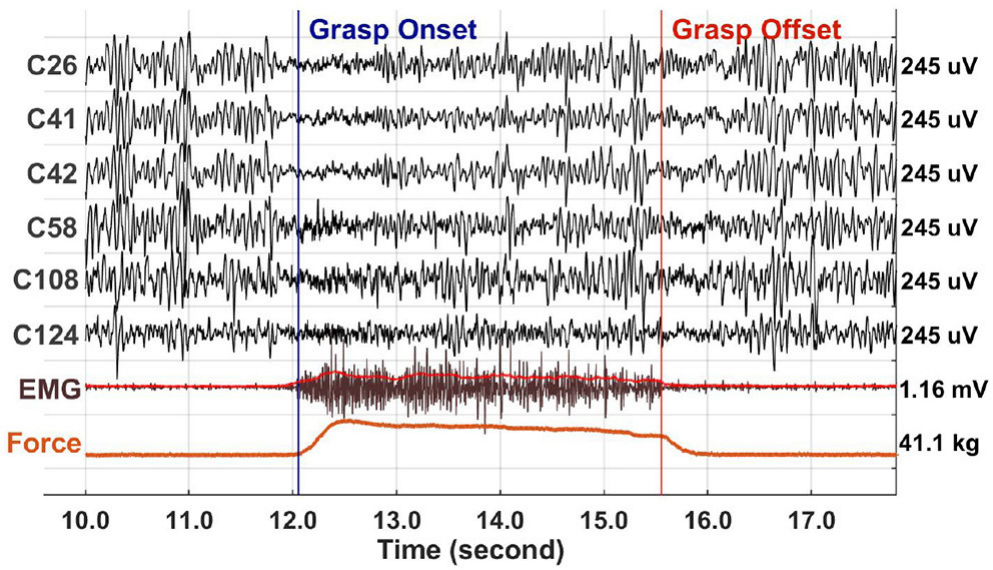
Single trial of synchronized data of ECoG, bipolar forearm EMG and hand grasp force (Force) for P2. The ECoG data were high-pass filtered at 3 Hz and forearm EMG data was high-pass filtered at 50 Hz for visualization. The RMS of rectified EMG is plotted in red over the EMG signal. The scale of each channel is shown on the right side. We can notice that the beta oscillations were suppressed during the onset and offset phases, but partially recovered in-between during the hold phase.

All ECoG recordings were visually examined for the identification of corrupted channels and artifacts. Three channels in P1, 24 channels in P2, 5 channels in P3, and 4 channels in P4 were corrupted and excluded from further analysis. The power line noise at 60 Hz and its harmonics were removed via a series of second-order infinite impulse response (IIR) notch filters. To perform event-aligned analyses, we used the minimum acceleration criterion with constraints (MACC) method to detect the beginning of grasp onset and offset on the root mean square (RMS) of EMG (Figure 2) (Botzer, 2009). MACC assumes a motor trajectory with two phases, a static phase followed by an active or movement phase. The model of the movement phase is based on a regression analysis that models the initial phase of the bell-shaped EMG power profile as a function of the cubic power of time (Botzer, 2009). This was successfully applied in our previous study to detect the onset of finger flexion and extension (Jiang et al., 2018). As in our recent study (Jiang et al., 2017), we executed a visual inspection using synchronized video, EMG and force sensor to ensure that a detected onset point was a real grasp execution onset rather than an artifact. We selected only trials with clean ramping EMG at grasp onset and without spontaneous EMG bursts over the 1.5 s baseline period before grasp onset. An epoch of ECoG data, forearm EMG, synchronized force, as well as grasp onset-offset from P2 data is shown in Figure 2. The peak force achieved by patients was less than the typical grip force for individuals of the same age and sex (Bohannon et al., 2006). The number of trials kept for analysis after visual inspection of the neural and behavioral data, as well as the video, is indicated in Table 1 for each patient and onset/offset phase.

### 2.4. Time-Frequency Analysis

In order to explore time-varying spectral dynamics of ECoG, data segments around grasp onset and offset (–1.5 to 1.5 s) were extracted and time-frequency analysis was performed at each channel using short-time Fourier transform (STFT). Specifically, the power spectral density (PSD) was estimated using a 512-sample Hanning window. The window was shifted with a 480-sample overlap for a smooth temporal transition.

Smoothed spectrogram of grasp onset and offset for each channel were obtained by averaging the spectrograms across trials:

(1) $$\begin{array}{r} {\bar{S}}_{c}^{t,f}=\frac{\sum_{m=1}^{Tr}S_{c,m}^{t,f}}{Tr} \end{array}$$

where *c* indicates the channel index, *t*(s) the time, *f*(Hz) the frequency, *m* the trial index, and *Tr* the total number of grasp onset or offset trials.

Baseline PSD ($R_{c}^{f}$) for channel *c* was estimated from the 500 ms of the spectrogram preceding the grasp onset (*t* = 0).

The average spectrograms for grasp onset and offset were normalized using the same baseline PSD and transformed into dB scale to yield the centered spectrograms (${Ŝ}_{c}^{t,f}$/${Ŝ}_{c}^{t,f}$). The normalization was performed through element-wise division along the dimension of frequency *f*.

(2) $$\begin{array}{r} {Ŝ}_{c}^{t,f}=10\log\frac{{\bar{S}}_{c}^{t,f}}{R_{c}^{f}} \end{array}$$

### 2.5. Event-Related Synchronization and Desynchronization Around Grasp Onset, Hold, and Offset

Event-related desynchronization (ERD) in the alpha and beta band (8–32 Hz) and event-related synchronization (ERS) in the gamma band (60–200 Hz) are well-known event-related neural modulations observed in various electrophysiological recordings at both cortical level (Pfurtscheller and Lopes da Silva, 1999; Pfurtscheller et al., 2003; Jiang et al., 2017, 2018), and subcortical level (Alegre et al., 2005; Brown and Williams, 2005; Kühn et al., 2006) during motor tasks. Specifically, ERS refers to the power increase, and ERD refers to power decrease during active periods relative to the reference period (Pfurtscheller and Lopes da Silva, 1999). We selected the 8–32 and 60–200 Hz frequency bands based on the visual inspection of the time-frequency maps and they are consistent with our previous studies (Jiang et al., 2017, 2018).

To compute the ERD and ERS of channel *c*, the original signal ($A_{c}^{t}$) was bandpass filtered in the low frequency band (LFB: 8–32 Hz) and the high frequency band (HFB: 60–200 Hz) using a second-order Butterworth IIR zero-phase filter (forward and backward). The filtered signals were squared to compute the power traces for LFB ($LP_{c}^{t}$) and HFB ($HP_{c}^{t}$):

(3) $$\begin{array}{r} LP_{c}^{t}=BP_{8-32Hz}{\left(A_{c}^{t}\right)}^{2} \end{array}$$

(4) $$\begin{array}{r} HP_{c}^{t}=BP_{60-200Hz}{\left(A_{c}^{t}\right)}^{2} \end{array}$$

The temporal evolution of ERD and ERS was computed by normalizing the power traces against their respective baseline power preceding the grasp onset.

In addition, ERD and ERS were estimated at grasp onset, hold and offset phases as follows. For grasp onset, they were computed from –0.1 to 0.7 s around the grasp onset. For grasp offset, they were estimated from the 0.8 s of data following immediately grasp offset. For the hold phase, we used 1–1.5 s of data segments in which the grip force reached a plateau. These segments were at least 1s away from the grasp onset and offset.

(5) $$\begin{array}{r} ERD_{LFB}=10\log(\frac{\sum_{t\in T_{phase}}LP_{c}^{t}}{\sum_{t\in T_{ref}}LP_{c}^{t}}\times \frac{N_{ref}}{N_{phase}}) \end{array}$$

(6) $$\begin{array}{r} ERS_{HFB}=10\log(\frac{\sum_{t\in T_{phase}}HP_{c}^{t}}{\sum_{t\in T_{ref}}LP_{c}^{t}}\times \frac{N_{ref}}{N_{phase}}) \end{array}$$

where *T*_*phase*_ is the time range defined for grasp onset, hold, or offset phase. *N*_*ref*_ and *N*_*phase*_ are the respective number of samples of reference and phase data.

The statistical significance of ERD/ERS at each channel was tested using a one-tailed Student's *t*-test with a significance threshold *p*-value of 0.05 and corrected for multiple comparisons using the False Discovery Rate (FDR) method at the level of 0.05 (Genovese et al., 2002). In order to select the most robust ERD and ERS events, we selected channels that had significant changes of at least 25% from baseline in either grasp onset or offset phase as previously done in (Jiang et al., 2018). For ERD, the alternative hypothesis (*H*_1_) was *ERD*_*LFB*_ <0.75 (–1.25 dB), while for ERS, *H*_1_ is *ERS*_*HFB*_>1.25 (+0.97 dB). The sample population of the *t*-test was the number of trials for each phase.

### 2.6. Spatial Patterns of LFB-ERD and HFB-ERS

We investigated the cortical spatial patterns of LFB-ERD/HFB-ERS around grasp onset and offset. To this end, we registered the position of the ECoG grid onto the 3D rendering of the brain computed from the individual preoperative MRI. The grid localization was determined by the neurosurgeons based on the coregistration of the intraoperative photograph of the cortex and the preoperative MRI scan (1 mm slice thickness) of the brain using bio-landmarks such as blood vessels, sulci, and gyri. Details of the method were described in our previous work (Jiang et al., 2018).

To compare the modulations anterior vs. posterior to the central sulcus, we averaged for each trial the spectrograms of channels with significant ERD or ERS on each side of the central sulcus. Linear Mixed Models (LMM) were used to analyze the effect of the grasp phase (onset, hold, offset), electrodes location (anterior, posterior), and their interaction on the power of LFB and that of HFB. The repeated measure covariance matrix was modeled using the first-order autoregressive structure. The analyses were performed using IBM SPSS (v. 25).

### 2.7. Correlation Between LFB-ERD/HFB-ERS and Force Yank

For each patient, we performed a cross-correlation analysis between the average time-series of LFB-ERD/HFB-ERS and the first time-derivative of grasp force (yank). The time-series were averaged across trials to improve the signal to noise ratio and yield a smooth estimation of the lag related to maximum/minimum cross-correlation coefficients. After that, the Pearson correlation coefficient was computed for individual trials by shifting LFB-ERD/HFB-ERS according to the identified lag from the averaged data. Force yank at sample k (*dF*_*k*_) was estimated using the numerical central difference on force (*F*): (*F*_*k*+1_−*F*_*k*−1_)/2. The correlation around grasp onset and offset were analyzed separately using the ECoG channels associated with significant LFB-ERD or HFB-ERS activations and using channels anterior or posterior to the central sulcus. Specifically, –1 to 1.5 s of data segment around grasp onset/offset was extracted to include also the hold phase.

Statistical analyses of lag and correlation were performed as follows. An LMM model was used to analyze the effect of the band (LFB-ERD, HFB-ERS), grasp phase (onset, offset), location (anterior, posterior), and their interactions on the lag between the time-course of yank and the time-course of band power. The repeated measures covariance matrix was modeled using the compound symmetry structure. Regarding the correlation, since we were interested in its strength rather than its direction we analyzed the absolute value of correlation. In addition, Fisher's z-transformation was applied to normalize the data. LMM was used to analyze the effect of the band (LFB vs. HFB), grasp phase (onset, hold, offset), location (anterior, posterior), and their interactions. The repeated measure covariance matrix was modeled using the first-order autoregressive structure. The analyses were performed using IBM SPSS (v. 25).

## 3. Results

### 3.1. LFB-ERD and HFB-ERS Magnitudes Between Anterior and Posterior Channels at Grasp Onset and Offset

**Figure 3:**
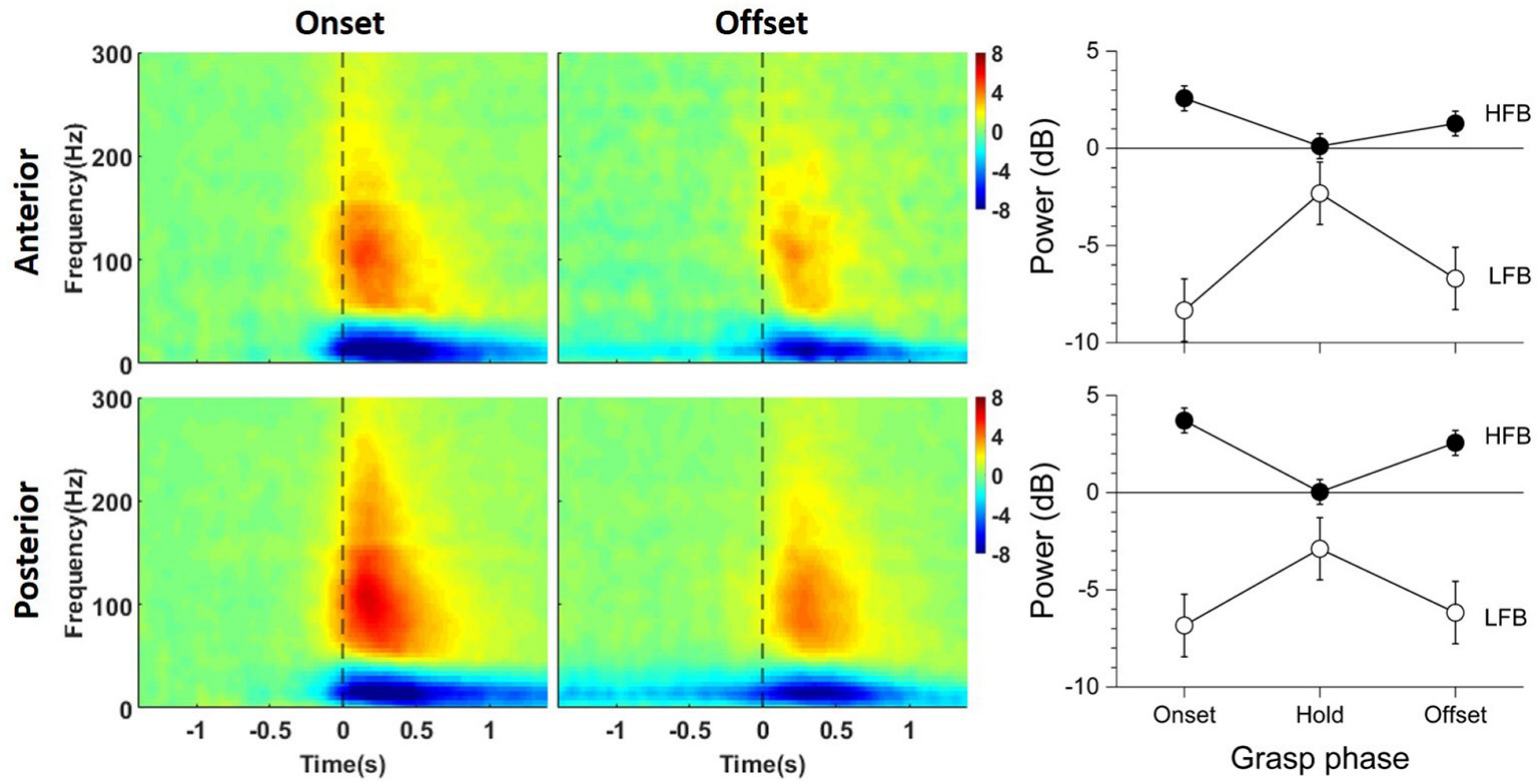
Average baseline-normalized time-frequency maps of significant channels anterior (top) and posterior (bottom) to the central sulcus around grasp onset (left) and offset (middle). On the right are the power deviation of LFB and HFB from baseline averaged across patients for each phase of the grasp. The error bars represent the 95% confidence interval.

Figure 3 shows the average time-frequency maps of channels anterior and posterior to the central sulcus around grasp onset and offset across patients. Two distinct power modulations, one in LFB (blue) and the other in HFB (red), were observed at both grasp onset and offset. Note that, although a sustained force level was maintained throughout the grasp hold phase, the ERD and ERS magnitude decreased and returned toward the baseline.

Overall, LFB-ERD lasted longer than HFB-ERS. The average time-frequency maps show that posterior channels tended to have larger grasp related modulations compared to anterior channels, especially for HFB-ERS at grasp onset. HFB-ERS and LFB-ERD from both anterior and posterior ECoG channels tended to slightly precede grasp onset. In contrast, HFB-ERS tended to lag grasp offset.

The right side of Figure 3 shows the average ERD/ERS for anterior and posterior channels during each grasp phase on the right side (See Supplementary Figure for the ERD/ERS levels of the individual subjects). The power of LFB was significantly different across phases of the grasp [*F*_(2, 656)_ = 82.311, *p* < 0.001], but not across locations [*F*_(1, 656)_ = 2.158, *p* = 0.142]. The power of LFB decreased during the onset and offset phase of the grasp, and returned close to baseline during the hold phase. Although close to the baseline, LFB remained significantly lower than the baseline, as the confidence intervals indicate (Figure 3). In addition, the grasp phase by location interaction was significant [*F*_(2, 656)_ = 3.273, *p* = 0.039]. The interaction occurred because the LFB-ERD was significantly stronger for anterior electrodes than posterior electrodes during the onset phase [*t*_(656)_ = –2.620, *p* = 0.009], but not during the other phases of the grasp [hold:*t*_(656)_ = 1.006, *p* = 0.315; offset: *t*_(656)_ = –0.922, *p* = 0.357].

The power of HFB also changed significantly across phases of the grasp, it increased during the onset and offset phases and returned to baseline during the hold phase [*F*_(2, 656)_ = 116.473, *p* < 0.001]. In addition, the power of HFB was significantly different across locations [*F*_(1, 656)_ = 23.413, *p* < 0.001], and the phase by location interaction was significant [*F*_(2, 656)_ = 6.882, *p* = 0.001]. The interaction occurred because the power of HFB was higher in the posterior electrodes than the anterior electrodes during the onset [*t*_(656)_ = –4.020, *p* < 0.001] and offset phases [*t*_(656)_ = –4549, *p* < 0.001] of the grasp, but not during the hold phase (*t*_(656)_ = 0.255, *p* = 0.799]. The power of HFB increased during the onset and offset phase of the grasp, and returned to baseline during the hold phase despite the sustained force level.

### 3.2. Spatial Patterns of ERD/ERS Around Grasp Onset and Offset

**Figure 4:**
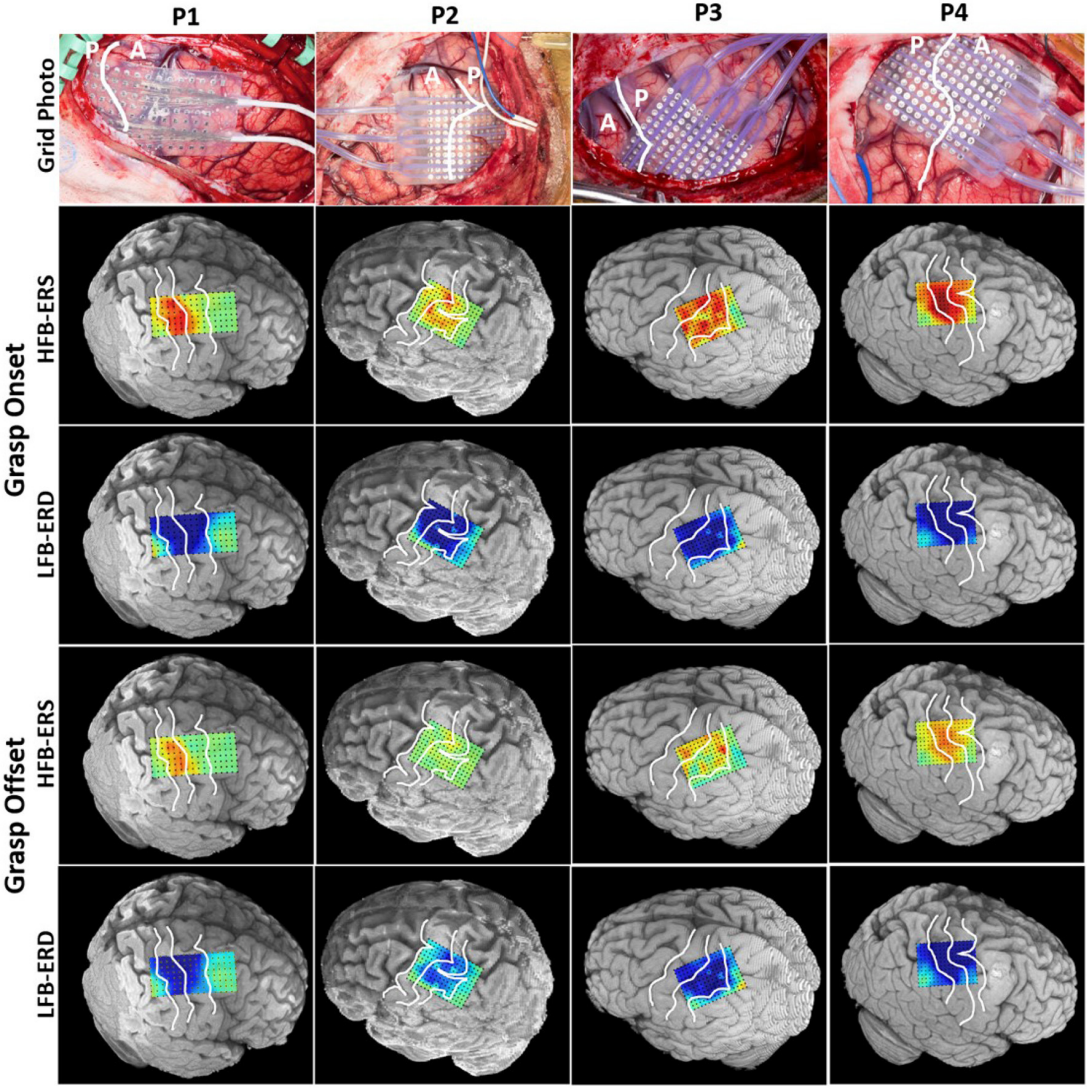
Intraoperative photograph of the grid placement shown for each patient in the first row. The central sulcus is marked with a white line (A: anterior, P: posterior). The photographs are in the same anteroposterior orientation as the 3D MRI brain models shown below. The spatial patterns of LFB-ERD (8–32 Hz) and HFB-ERS (60–200 Hz) around grasp onset and offset phases were mapped onto the individual 3D MRI rendering of the cortex for each patient. The three white lines on each MRI brain model identify the precentral sulcus, central sulcus, and postcentral sulcus. All power maps were scaled from –6 to 6 dB.

The spatial patterns of LFB-ERD (blue) and HFB-ERS (red) around grasp onset and offset were mapped onto 3D rendering of the brain computed from the individual preoperative MRI (Figure 4).

The HFB-ERS patterns tended to follow the anatomic boundaries (precentral, central, and postcentral sulci) in all patients. Most of the significant HFB-ERS channels were found to be within the primary somatosensory cortex. In contrast, the significant LFB-ERD channels were widely distributed on both sides of the central sulcus. Overall, LFB-ERD had a broader spatial distribution than HFB-ERS. Fewer significant LFB-ERD and HFB-ERS channels were found generally at grasp offset compared to grasp onset.

### 3.3. Temporal Evolution of ERD/ERS in Regards to Force and Force Yank

**Figure 5:**
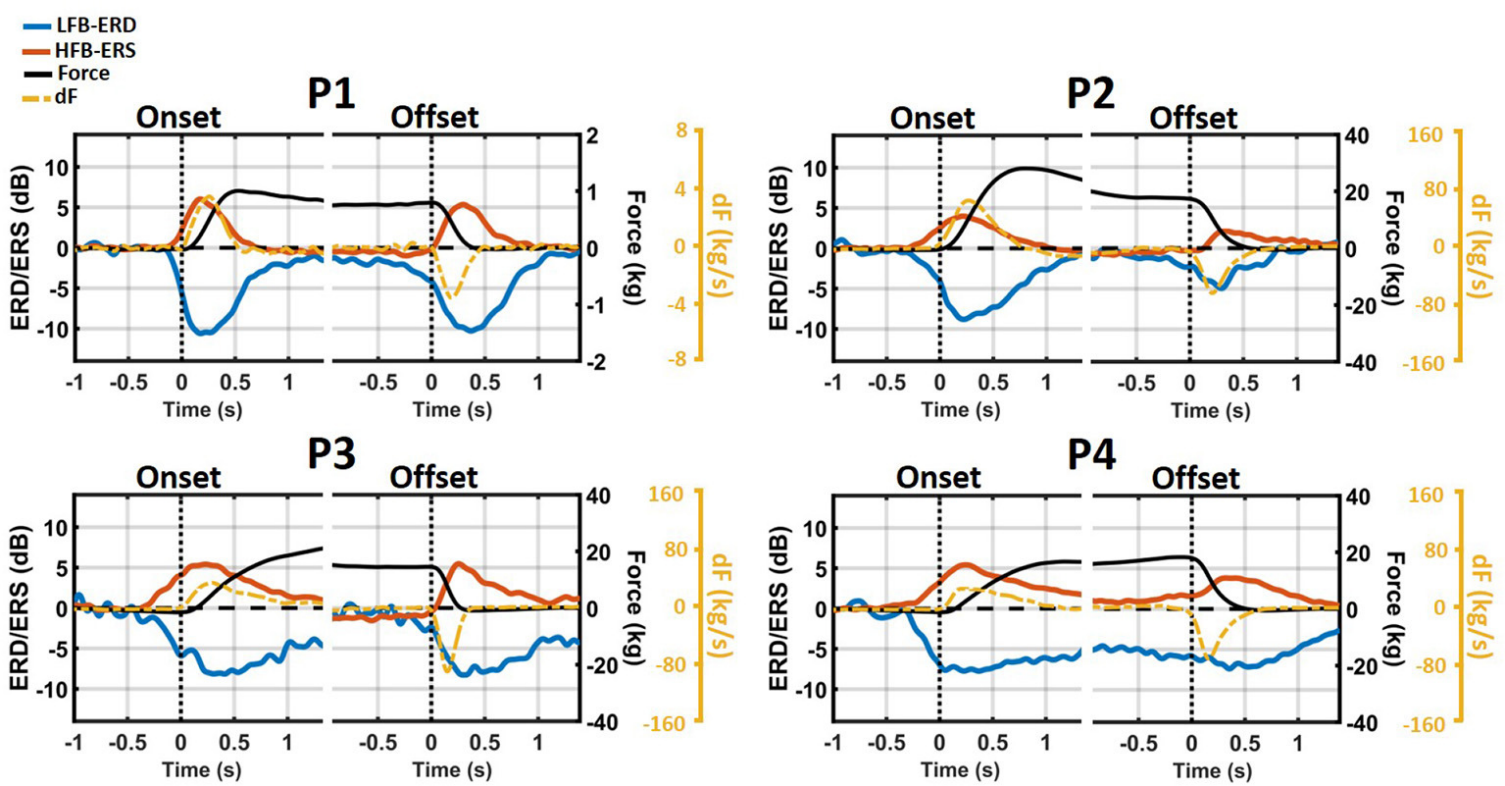
Temporal evolution of average LFB-ERD (blue), HFB-ERS (red), grip force (black), and the first time-derivative of force (dF, orange) around grasp onset and offset phases. The ERD/ERS were averaged across all significant channels and displayed from –14 to 14 dB for all patients while the scales of Force and dF are different across patients and provided on the right side of each figure. Both LFB-ERD and HFB-ERS started between 100 and 300 ms prior to the onset of the grasp.

The temporal evolution of average LFB-ERD (blue) and HFB-ERS (red) around grasp onset and offset is shown together with the average force trace (black) in Figure 5. As can be seen from the average force levels of individual subjects, the grip force reached a plateau. In addition, the time-course of force yank (dF) is shown as a dashed orange line. Due to the difference in the amount of force exerted by the patients, force and its derivative were scaled separately for each one of them. The ERD/ERS traces and time-frequency maps were scaled the same (–14 to 14 dB) for all patients.

In all patients, LFB decreased and ERS increased during the onset and offset phases of the grasp. However, despite that a sustained force level was maintained throughout the hold phase, the magnitude of LFB-ERD and HFB-ERS decreased and returned at or close to the baseline level.

Both LFB-ERD and HFB-ERS preceded grasp onset by 100 to 300 ms. However, the peaks of both subband modulations occurred after grasp onset but near the time of the peak of force yank. At grasp offset, however, ERS generally started right at or after the beginning of the relaxation phase. In contrast, LFB-ERD generally started before the relaxation phase. The peaks of both LFB-ERD and HFB-ERS occurred after the peak of force yank, that is, 200–300 ms after the start of the relaxation phase.

Although neither the profile of the time-changing LFB-ERD nor that of HFB-ERS resembled the time-course of force, both were similar in absolute value to the time-course of force yank. Especially during grasp onset, HFB-ERS closely matched force yank for all patients. The similarities between ERD/ERS and force yank during grasp offset were not as high as during grasp onset. Overall, at grasp onset, the correlation between HFB-ERS and force yank (*r* = 0.79±0.03) was significantly stronger than with raw force [*r* = 0.31±0.2; paired *t*_(3)_ = 10.1, *p* = 0.002]. The correlation between HFB-ERS and force yank was slightly weaker at grasp offset and was not significantly different than the correlation with the raw force [yank: *r* = −0.61±0.1; raw force: *r* = −0.63±0.1; paired *t*_(3)_ = 0.23, *p* = 0.83]. At grasp onset and offset the correlation between LFB-ERD and force yank was stronger (onset: *r* = −0.6±0.08; offset: *r* = 0.35±0.17), than the raw force (onset: *r* = 0.44±0.05; offset: *r* = 0.24±0.4), and weaker compared to the HFB-ERS.

**Figure 6:**
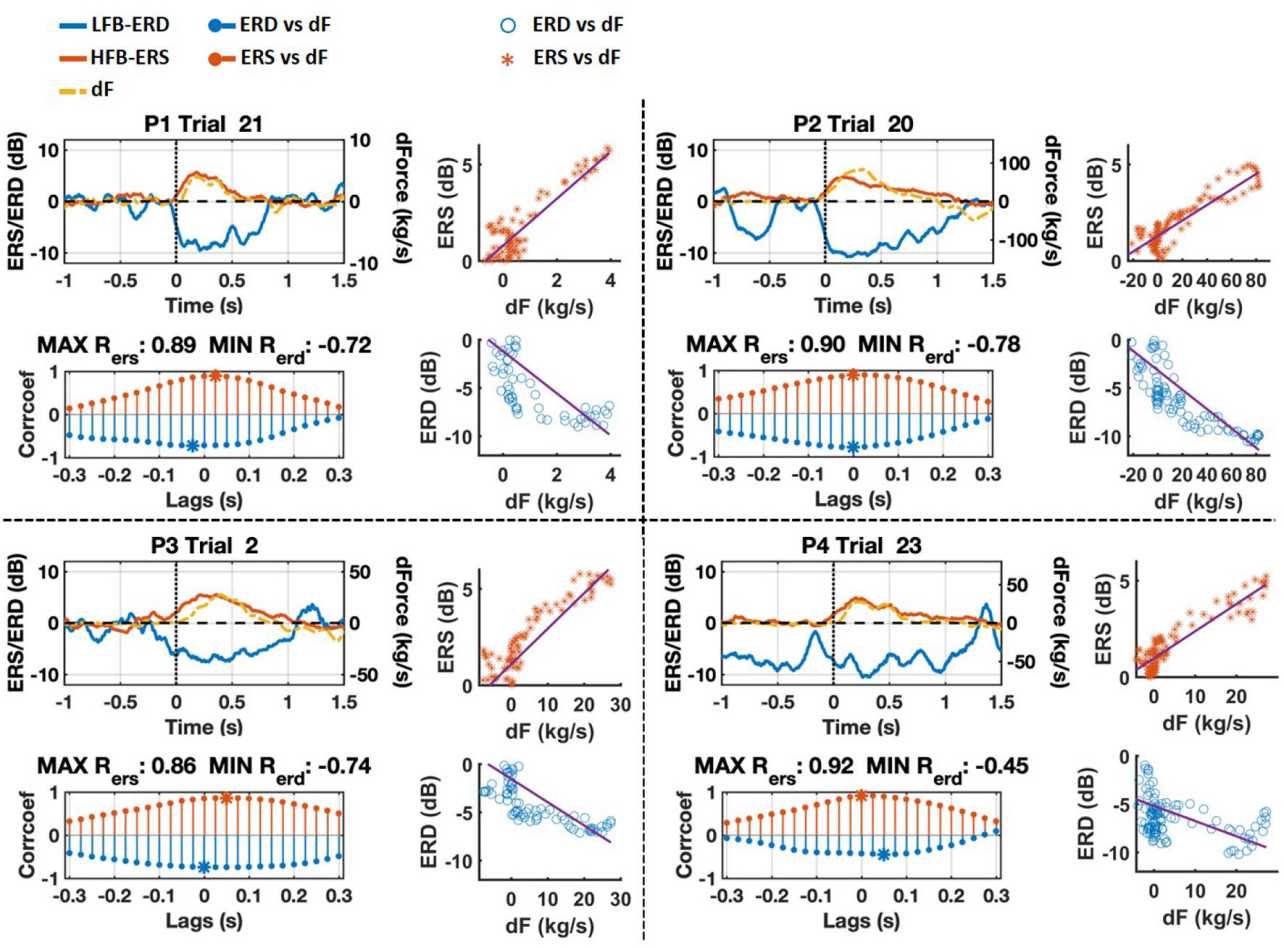
Grasp onset phase of trials with maximum cross-correlation between HFB-ERS and force yank (dF) for each patient. The scale of force gradient was adjusted for each patient and is shown on the right side. ERD and ERS are scaled between –12 and 12 dB. Cross-correlation results with respect to lags between –0.3 and 0.3 s are also shown for each trial. The strongest correlation points are marked by asterisks. The scatter plots of ERS/ERD vs. dF of selected trials shifted according to maximum cross-correlations are shown on the right side as well.

The trial with maximum cross-correlation coefficient between HFB-ERS and force yank around grasp onset for each patient is shown in Figure 6 (P1: trial 21, P2: trial 20, P3: trial 2, and P4: trial 23). The correlation between HFB-ERS and force yank was positive at grasp onset whereas it was negative for LFB-ERD. The maximum correlation coefficient for the trials illustrated and the corresponding lag between HFB-ERS and force yank for each patient was, P1: 0.89 (25 ms), P2: 0.90 (0 ms), P3: 0.86 (50 ms), P4: 0.92 (0 ms). The correlation coefficient between LFB-ERD and force yank for the same trial was, P1: –0.72 (–25 ms), P2: –0.78 (0 ms), P3: –0.74 (0 ms), P4: –0.45 (50 ms). It is clear from both the time-course plots and scatterplots that LFB-ERD and HFB-ERS closely followed the time-course of force yank. In addition, the correlation of force yank with HFB-ERS was stronger than the correlation with LFB-ERD. Based on these observations we performed additional cross-correlation analyses.

**Figure 7:**
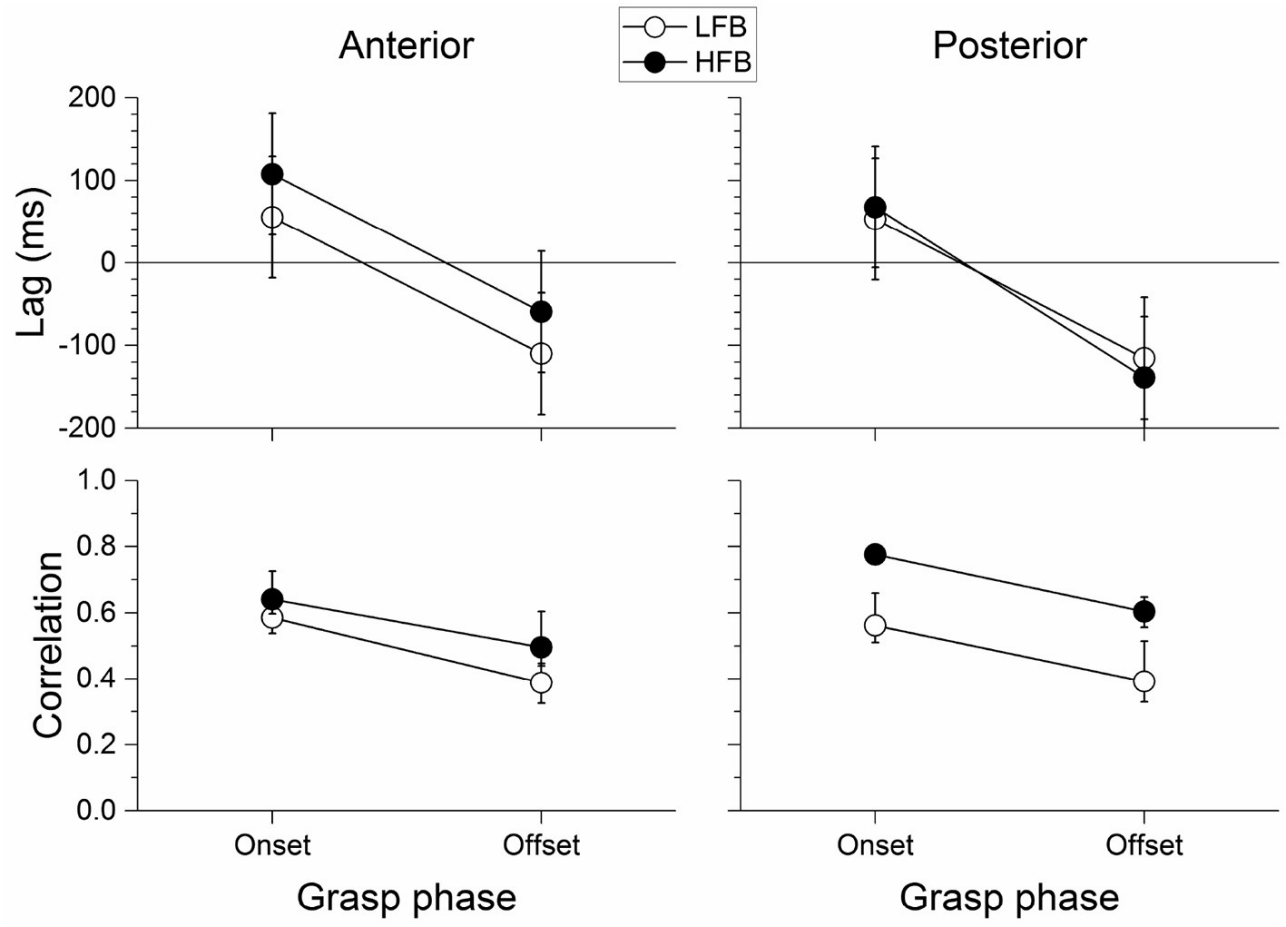
Average lag (top) and absolute correlation coefficient (bottom) between neural activity and force yank around grasp onset and offset phases for anterior (left) and posterior (right) significant channels. Correlations were averaged using Fisher z-transformation, and then transformed back in correlation scale. Error bars represent the 95% confidence interval. The lag between neural activity and force yank was positive at the onset and negative at the offset indicating that the neural activity was leading and then lagging the force yank during the onset and offset phases, respectively. The correlation between LFB/HFB power and force yank was stronger during the onset than the offset phase of the grasp. In addition, the posterior channels were significantly more correlated with force yank than the anterior channels, and particularly so for HFB.

In Figure 7 (top) we show the average lag between the time-course of force yank and that of LFB-ERD and HFB-ERS for electrodes anterior and posterior to the central sulcus. Positive lag indicates that changes in LFB or HFB preceded force yank, whereas a negative lag indicates the reverse. The lag was significantly different between grasp phases [*F*_(1, 24)_ = 49.535, *p* < 0.001]. It was on average 71±37 ms for the onset phase and −106±72 ms for the offset, that is, the time-course of power led yank at the onset but lagged at the offset phase. No other effect of band, location, or interactions reached statistical significance. In particular, although the lag between the HFB-ERS and force yank was larger in the anterior (108±44 ms) than the posterior region (68±40 ms) at the grasp onset, where the neural activity led the changes in force, this difference was not significant [*t*_(24)_ = 1.346, *p* = 0.191].

Figure 7 (bottom) shows the absolute correlation between force yank and LFB-ERD or HFB-ERS at those lags. The correlation was significantly different between bands [*F*_(1, 840)_ = 107.880, *p* < 0.001], grasp phase [*F*_(1, 840)_ = 141.500, *p* < 0.001], and location [*F*_(1, 840)_ = 21.283, *p* < 0.001]. In addition, the band by location interaction was also significant [*F*_(1, 840)_ = 28.678, *p* < 0.001]. No other interaction reached significance. The correlation was stronger for the HFB than for the LFB. It was also stronger during the onset phase of the grasp than during the offset phase. In addition, the correlation for posterior HFB was stronger than for posterior LFB, whereas the difference was much smaller between the correlation for anterior HFB and anterior LFB.

## 4. Discussion

Previous ECoG BMI studies on hand grasp have mainly focused on the decoding of movement kinematics, such as finger positions, grasp aperture, and velocity (Kubánek et al., 2009; Miller et al., 2009; Acharya et al., 2010; Pistohl et al., 2012; Nakanishi et al., 2014; Flint et al., 2017). The few studies that investigated the decoding of the kinetic aspects of hand grasp, especially gripping force, often used grasp tasks with relatively short (<0.1 s) or no explicit holding period (Chen et al., 2014; Flint et al., 2014). In this study, intraoperative high-density ECoG was recorded from four patients while they were instructed to execute sustained hand grasps (>2 s) during awake surgeries without any timing pressure. They were simply guided during the task and were not instructed to move as quickly as possible. We found consistent sensorimotor phasic subband modulations during the grip onset and offset phases but the hold period. In particular, both LFB-ERD and HFB-ERS started 100 to 300 ms before grasp onset. During the holding period, the magnitude of LFB-ERD and HFB-ERS decreased markedly although grip force was maintained. Nevertheless, across patients, LFB-ERD remained significantly below baseline during the hold phase. In contrast, during that same period, HFB-ERS was not significantly different than baseline. At grasp offset, while the LFB-ERD started earlier than the relaxation phase, the HFB-ERS lagged it. They both peaked after 200 to 300 ms following the beginning of the relaxation phase. The modulations lasted throughout the relaxation phase, and even after the movement had ceased.

### 4.1. Temporal Dynamics of LFB-ERD and HFB-ERS

Previous studies have shown that internally and externally cued movements were associated with partially distinct spatial and temporal ERD and ERS patterns (Tsang et al., 2012; Pereira et al., 2018). Tsang et al. have shown that externally cued movements were associated with beta ERD and gamma ERS which started at the onset of the movement, whereas self-initiated movements were associated with premovement ERD and ERS (Tsang et al., 2012). However, in our study, both LFB-ERD and HFB-ERS started 100 to 300 ms before grasp onset. This could be related to the fact that although patients were asked to execute the sustained hand grasp, they initiated the movement without any time constraint.

In our study, despite that, a sustained force level was maintained throughout the hold phase, in all subjects, the absolute magnitude of LFB-ERD and HFB-ERS consistently decreased during the hold period. Except for P3, although close, in 3 out of 4 subjects the LFB-ERD was significantly lower than the baseline (see Supplementary Material). In patients P3 and P4, HFB-ERS returned to the baseline during the hold period. While P2 had significantly higher HFB-ERS, P1 had significantly lower HFB-ERS than the baseline. Compared to other subjects, for P4, the LFB-ERD decreased but it did not return close to the baseline. The HFB-ERS level was also slightly above the baseline and had a large variance. These deviations from the baseline and large variance in HFB-ERS could be due to the fact that this particular subject did not keep the grip force at a very steady level and had a slightly elevated force amplitude toward the end of the hold period.

Remarkably, in their pioneering ECoG studies, Jasper and Penfield had also illustrated the phasic changes of motor cortical LFB during the onset and offset phases of hand clenching (see their Figure 5) (Jasper and Penfield, 1949). Szurhaj et al. reported that stereoelectroencephalography (SEEG) channels exhibited low gamma ERS (40–60 Hz) related to either grasp onset or offset but never in both (Szurhaj et al., 2005). In contrast, Ball et al. reported in their center-out/center-in arm reaching experiments that a few ECoG channels were associated with high gamma (HG) band (50–150 Hz) ERS at grasp onset and offset (Ball et al., 2008). These differences between studies could be due to the disparity of cortical area sampled in the SEEG study (disjoint sites, 62–92 deep electrode) vs. the ECoG study (continuous sites, 112 contacts with 7.1 mm inter-electrode distance). A study of sustained hand grasp in non-human primates showed distinct peaks of gamma modulations at both grasp onset and offset (Waldert et al., 2015). Others also briefly mentioned the presence of biphasic peaks of ERS in human ECoG during hand grasp (Flint et al., 2017). Interestingly, recent work on the response to deep, light and soft touch (Kramer et al., 2019) with ECoG showed that elevations in HG power seen within selected electrodes over the hand area of primary somatosensory cortex (S1), lasted between around 300 and 500 ms, but extinguished prior to the end of the tactile stimulus. This coincides with our findings regarding the S1 high-gamma attenuation pattern during the hold period of a grasp task and Figure 3 shows that high-intensity gamma response (50–150 Hz) peaked between 50 and 400 ms after grasp onset, within selected electrodes anterior and posterior to the central sulcus.

### 4.2. Connections to the Dynamics of the Afferent System

During grasp onset, finger movements and the pressure exerted on the hand dynamometer activates not only the muscle spindles but also the slowly-adapting, low-threshold mechanoreceptors, such as the Merkel cell-neurite complex found in the basal layer of the epidermis of fingers, as well as the slowly-adapting Ruffini endings broadly expressed in the dermis. Merkel cells respond to deep touch or pressure with current decay of ~200–300 ms (Roudaut et al., 2012). The Ruffini endings respond to stretch of the skin and pressure with current decay of ~1000 ms (Roudaut et al., 2012; Delhaye et al., 2018). The handling of the hand dynamometer also stimulates the rapidly-adapting Meissner corpuscles located in the dermal papillae of the glabrous skin as well as the rapidly-adapting Pacinian corpuscles, by way of skin deformation and indentation (Roudaut et al., 2012; Delhaye et al., 2018). The slowly adapting receptors generate an initial burst of action potentials where their rate decays slowly over time (200–300 ms for Merkel cells and ~1000 ms for Ruffini endings). In contrast, any physical deformation of the Meisner corpuscles triggers a burst of action potentials that quickly ceases, and when the stimulus is removed, the corpuscles regain their shape and while doing so produce another volley of action potentials (Roudaut et al., 2012; Delhaye et al., 2018). In a recent study, Ryun et al. showed that the HG response to tactile stimulation (course and fine texture stimulation) mimics the behavior of the Meissner and Pacinian corpuscles immediately after the stimulation onset and offset (Ryun et al., 2017b). This phasic activation of fast adapting receptors is consistent with the HFB-ERS and LFB-ERD at grasp onset and offset that we observed. It is likely that the late HFB-ERS at grasp offset is associated with the burst activity of fast adapting Meissner and Pacinian corpuscles due to the release of stimulation and skin deformation recovery. Similar phasic activity of spiking neurons was found in rats' primary sensory cortex at the onset and offset phases of a sustained touch task (Choi et al., 2016).

Interestingly, HFB-ERS was mostly at the lower end of the high gamma spectrum (60–200 Hz) during the hold period. A similar phenomenon had been found in our previous study on hand flexion/extension (Jiang et al., 2017). Others reported that low gamma ERS (40–60 Hz) often started at or after grasp onset and could last longer than the movements, suggesting that it may be linked to a combination of movement execution and sensory feedback related modulations (Crone et al., 1998; Szurhaj et al., 2005). Together with phasic response of fast adapting receptors, it is likely that the fast firings generated by the slowly adapting Ruffini endings and Merkel cells contribute to the early high-intensity broadband gamma response that we observed (Figure 5) by way of skin deformation, indentation, as well as pressure at the onset of the grasp (Roudaut et al., 2012; Ryun et al., 2017b; Delhaye et al., 2018; Kramer et al., 2019). The broadband high-frequency ERS later reduces to a low-intensity gamma response at a lower frequency as time progresses and this behavior can be related to the activation patterns of the slowly adapting receptors of the afferent system (Roudaut et al., 2012; Delhaye et al., 2018).

### 4.3. Possible Modulations Due to Efferent System

We observed HFB-ERS both during the onset and offset of the grasp but not during the hold period. Interestingly the HFB-ERS started earlier before the onset of the grip force in both motor and sensory areas. Whereas, later, during the offset of the grasp, the HFB-ERS lagged the changes in force. Given that the HFB-ERS preceded grasp onset and changes in the force in both the primary motor cortex (M1) and the primary somatosensory cortex (S1), it is difficult to relate the observed HFB-ERS to the pressure dynamics of the afferent system only. We presume that the modulations that we observed during the onset of the grasp are likely a mixture of both efferent and afferent systems. However, during the offset of the grasp, the HFB-ERS generally lagged the changes in force. Therefore, it is likely that this late modulation is a result of the pressure dynamics of the afferent system.

LFB-ERD started earlier than the grip onset suggesting that LFB-ERD is associated with the initiation of the movement. Interestingly, while LFB-ERD started earlier, the HFB-ERS lagged the grip offset. Since LFB-ERD preceded both the onset and offset of the grasp and peaked after each, it is clear that the 8–32 Hz range is modulated by the efferent system at the start of the movement, but it is possible that it is also modulated by the afferent system during the movement.

Consequently, the results indicate that the initiation and termination of grasp are associated with distinct neural activity sources whereas the HFB-ERS represents the dynamics of the afferent and efferent systems at the grip onset, but probably only the afferent system at the offset that corresponds to the relaxation phase. Since gamma oscillations are related to the activity of local circuits (Buzsáki and Wang, 2012), the lack of HFB-ERS preceding grip termination brings the question of whether the sensorimotor areas are involved in the termination of movement in a different way to the initiation. Transitioning to the grasp and relaxation phases are not the same processes in opposite directions. The gripping phase requires control of force to increase it up-to when a certain level is reached, which rests on the coordinated control of finger, hand and arm muscles. There is no opposite equivalent for the relaxation phase, in that the relaxation does not require control of force until a no force is reached. There is also no need to finely coordinate finger, hand and arm muscles to relax the hand. The offset simply represents a disengagement from the hold period of the grip. All in all, the gripping phase does require more control than the relaxation phase, which may explain why there is a less clear signature of neural involvement before the relaxation phase. Interestingly, in their pioneering work, Smith et al. showed that neurons recorded from the primary motor cortex of non-human primates were majorally modulated during the dynamic phase of a sustained precision grip rather than during the static phase (Smith et al., 1975). Moreover, while a large portion of neurons started to discharge during the onset and offset phase of the grip task, the activations preceded the onset and lagged the offset of the grasp as we observed with the HFB-ERS modulations. These results suggest that HFB-ERS and motor cortical neurons show a similar phasic and temporal profile during the different phases of the grip and the onset and offset phases are associated with different neural signatures.

### 4.4. Spatial Profile of HFB-ERS and LFB-ERD

Although similar ERD/ERS patterns were observed in ECoG channels anterior and posterior to the central sulcus we found that HFB-ERS was generally more extended, and of greater amplitude in posterior channels than anterior ones. The level of HFB-ERS and its correlation with force yank was stronger in S1 compared to M1. In addition, in regards to the relation between the time-course of HFB-ERS and force yank, while both preceded the force yank, the anterior channels generally preceded posterior channels. However, this difference was not significant. Additionally, even though all ECoG channels had a lead over force yank during the onset phase of the grasp, most lagged during the offset phase. In summary, there were modest differences between ECoG channels anterior and posterior to the central sulcus, and the differences changed to some extent depending on the phase of the grasp. In a recent study (Ryun et al., 2017a), it was also discovered that HG activity in S1 was more dominant than in M1 during active, voluntary movement. Others have also confirmed that humans' sensory information is present in M1 recordings, in addition to motor responses in S1 (Schieber and Hibbard, 1993; Sanes et al., 1995; Schroeder et al., 2017). In the ECoG study with individual finger decoding (Hotson et al., 2016), there was a large amount of overlap between channels used for motor and sensory tasks, with the majority of the electrodes used being postcentral in both cases.

One may have expected greater differences on the basis of a simplistic view of the motor cortex and somatosensory cortex, as associated exclusively with motor commands vs. sensory signals, respectively. However, motor control of grasping, like that of other voluntary movements, is regulated directly by motor commands, and indirectly by the modulation of sensory afferents (Ueno et al., 2018). In particular, these regulations rests on corticospinal projections, including corticomotoneuronal projections, originating from both M1 and S1 (Coulter and Jones, 1977; Rathelot and Strick, 2006; Matyas et al., 2010; Ueno et al., 2018). In addition, M1 and S1 are reciprocally connected (Kunzle, 1978; Arce-McShane et al., 2016). For these reasons, it may not be entirely surprising that voluntary movement-related oscillatory activity from M1 and S1 share similar characteristics and have relatively small quantitative differences.

On the other hand, there were even fewer differences for LFB-ERD, which tended to have similar characteristics in anterior and posterior ECoG channels. Due to the limitations of electrical recording, it is possible that the wide-spread LFB-ERD suffered from volume conduction. Freeman et al. pointed out that the optimal inter-electrode spacing to avoid aliasing for ECoG is 1.25 mm (Menon et al., 1996). Another study using high-density ECoG with 1.5 mm contact size and 4 mm inter-electrode distance has shown to have significantly lower coherence in 60–120 Hz range, indicating non-redundant recordings for the gamma band (Menon et al., 1996). We and others have previously shown that the low and high-frequency oscillations of ECoG are associated with different spatial correlation levels. Recently, Rogers et al. (2019) showed that the correlation between recording contacts of ECoG electrodes drops quickly for high frequencies after 2–3 mm, but takes longer to drop for lower frequencies. In our earlier work, we recorded ECoG during resting and movement execution with electrodes having 1.2 mm contact size and 4 mm spacing (Jiang et al., 2015). Similarly to Rogers et al., in the resting state, we observed that the 8–32 Hz band overlapping with alpha-beta oscillations were associated with much higher spatial correlations compared to the gamma oscillations 60–200 Hz. During the movement execution phase, the correlation dropped in ERD and ERS associated areas. The drop in correlation within the 8-32 Hz band may be due to the amplitude suppression of alpha and beta oscillations (alpha/beta-ERD) during movement. Although the movement is associated with amplitude enhancement in gamma band (gamma-ERS), these high-frequency oscillations were still weakly correlated between neighboring channels. Since we used ECoG grids with around 1 mm contact exposure and 3–4 mm spacing, the similar characteristic of HFB-ERS between anterior and posterior channels can not be explained with volume conduction. However, the similarity in widespread LFB-ERD could be due to volume conduction or broader network synchronization in sensorimotor areas in the resting state.

### 4.5. Possible Challenges in ECoG Decoding for Sustained Hand Grasp

Published studies on decoding the force of hand grasp generally assumed a linear relation between brain oscillatory activity, such as beta and gamma-band power of ECoG or LFP, and grasp force (Chen et al., 2014; Flint et al., 2014; Milekovic et al., 2015; Tan et al., 2016). However, in this study, we show that the dynamics of LFB-ERD and HFB-ERS were more congruent with the first time-derivative of force rather than force itself. In spite of a wide spectrum of force generated (1 to 30 kg), significant correlations between the time-course of LFB-ERD/HFB-ERS and the first time-derivative of force (yank) were found across all four patients. At grasp onset and offset, the correlation levels between HFB-ERS and force yank were significantly higher in posterior channels compared to anterior channels. Compared to HFB-ERS, LFB-ERD had weaker correlations with force yank. At grasp offset, the correlation between HFB-ERS/LFB-ERD and force yank was inverted and both neural activations lagged the force yank. In addition, the correlation between HFB-ERS and force yank was smaller for both anterior channels and posterior channels at grasp offset compared to grasp onset.

Due to the fact that both HFB-ERS and LFB-ERD dramatically decreased at or near baseline level during the hold period, previous approaches for the decoding of grasp force utilizing ERS/ERD would fail in cases of sustained grasp, such as studied here, although they may work for “pulse”-like grasp movement without prolonged holding period (Flint et al., 2014). Moreover, the lack of HFB-ERS patterns forecasting the transition to the relaxation at the offset of the grasp will likely add challenges in decoding oscillatory neural activity involving sustained hand grasp. It is likely that information across multiple frequency bands and non-linear dynamics of oscillatory activity need to be integrated to accurately decode the state transitions and the details of the hand grasp.

## 5. Conclusions

Understanding the neural encoding of force strength during a hand grasp task is important for the development of neuroprostheses for common daily motor activities. In this study, we recorded high-density ECoG intraoperatively from the sensorimotor cortex of four patients. Patients were instructed to execute and maintain an isometric hand grasp for 2 to 3 s during awake craniotomies. Two distinct peaks of subband power modulations in the form of ERD in LFB (8–32 Hz) and ERS in HFB (60–200 Hz) were found within the primary motor and primary somatosensory cortices consistently across all four patients around the time of grasp onset and offset. Although the grasp force was maintained during hold, the magnitude of LFB-ERD and HFB-ERS decreased toward the baseline. Consistently in all patients, we show that the dynamics of gamma ERS and beta ERD were correlated with the first time-derivative of force (yank) rather than with force itself. Particularly, HFB-ERS had stronger correlations with force yank than LFB-ERD. In addition, HFB-ERS was found to be distributed with larger intensity and spatial extent on the posterior side of the central sulcus compared to the anterior side. In general, due to the biphasic characters of HFB-ERS/LFB-ERD at grasp onset and offset, force decoding algorithms based on the cortical oscillatory activity should be carefully designed to preserve the memory of the system. Future strategies aimed at decoding sustained grasp force from subband modulations will need to model the first-time derivative of grasp force in order to develop useful neuroprostheses.

## Data Availability Statement

The data that support the findings of this study are available on reasonable request from the corresponding author. The raw data are not publicly available due to data might contain potentially identifying or sensitive information that could compromise the privacy of research participants.

## Ethics Statement

The studies involving human participants were reviewed and approved by the Institutional Review Boards (IRB) of MD Anderson Cancer Center and the University of Houston. The patients/participants provided their written informed consent to participate in this study.

## Author Contributions

NI designed the study. NI, TJ, and PA collected the data. TJ, NI, and GP conducted the analysis. TJ, NI, GP, and PA wrote the manuscript and interpreted the results. SP and DB performed the surgeries. SP, DB, and ST contributed to data collection, imaging, and interpretation of the results. ST and SB performed the behavioral tests during surgery, evaluated the condition of subjects. All authors reviewed the manuscript and approved the final version of the manuscript.

### Conflict of Interest

The authors declare that the research was conducted in the absence of any commercial or financial relationships that could be construed as a potential conflict of interest.
