# Supplementary material for: Power Modulations of ECoG Alpha/Beta and Gamma Bands Correlate With Time-Derivative of Force During Hand Grasp
Source: Front Neurosci. 2020 Feb 14;14:100. doi: 10.3389/fnins.2020.00100 (PMC7033626; doi:10.3389/fnins.2020.00100)
Supplement: Supplementary file 1 [file Data_Sheet_1.PDF]

Supplementary Material

1 TIME-FREQUENCY ANALYSIS OF INDIVIDUAL PATIENT

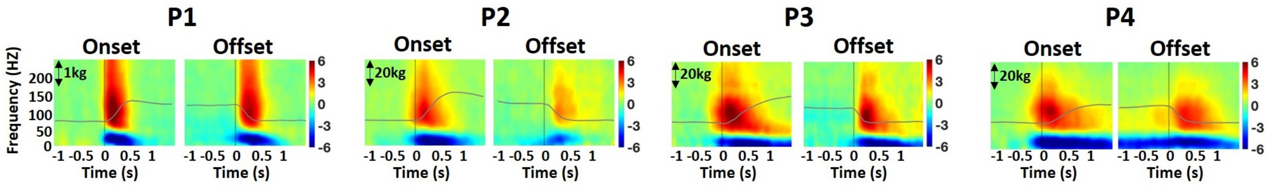

Figure S1: Centered time frequency maps averaged across channels with significant ERD and ERS during grasp onset and offset for each patient. Each time-frequency map covered -1 to 1.25 s around the onset or offset with a spectral range of 0 to 250 Hz. All maps are displayed from -6 to 6 dB. The time-varying grip force is superimposed on each onset and offset map as a dark line. The scale of force is provided on the top left corner for each patient.

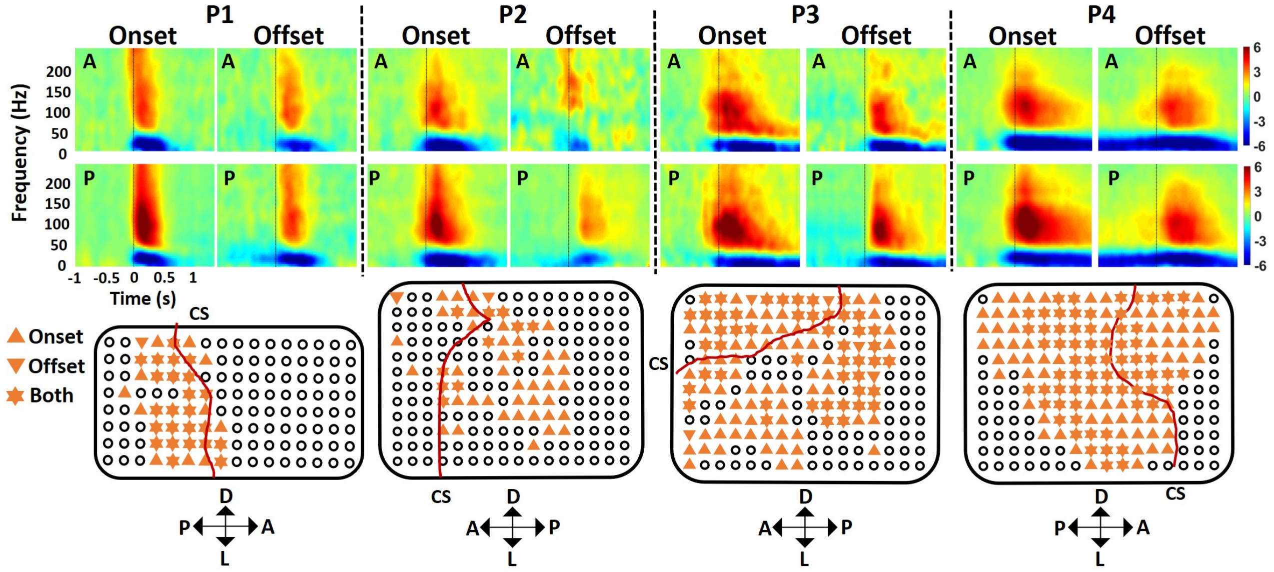

Figure S2: Centered time frequency maps averaged across significant channels anterior (A) or posterior (P) to the central sulcus are shown around grasp onset and offset for each patient. Each time-frequency map covered -1 to 1.25 s of the grasp onset/offset with a spectral range of 0 to 250 Hz. All maps are displayed from -6 to 6 dB. The channels used for averaging were marked on the grids below with upper triangles denoting significant channels during grasp onset, downward triangles denoting channels significant during grasp offset, and stars denoting channels significant during both grasp phases. The location of the central sulcus is marked by a red line on each electrode grid. The orientation of each grid is indicated at the bottom (A: anterior, P: posterior, D: dorsal, L: lateral).

The time-frequency maps averaged across channels around grasp onset and offset are shown for each patient in Figure S1. For all four patients, two distinct power modulations, one in LFB (8-32Hz, blue) and the other in HFB (60-200Hz, red), can be observed at both grasp onset and offset. Note that, although a sustained force level was maintained throughout the trial, the ERD and ERS magnitude decreased and returned towards baseline between grasp onset and offset. Overall, LFB-ERD lasted longer than HFB-ERS for all patients. LFB-ERD in P1 and P2 decreased close to baseline level after grasp onset, whereas for P3 and P4, LFB-ERD lasted throughout the hold period with only a slightly decreased magnitude. Figure S2 shows for each patient the average time-frequency maps of channels anterior and posterior to the central sulcus around grasp onset and offset. Channels show significant activations at either grasp onset or offset were annotated on the grids in Figure S2.

2 LFB-ERD AND HFB-ERS LEVELS OF GRASP ONSET, HOLD AND OFFSET

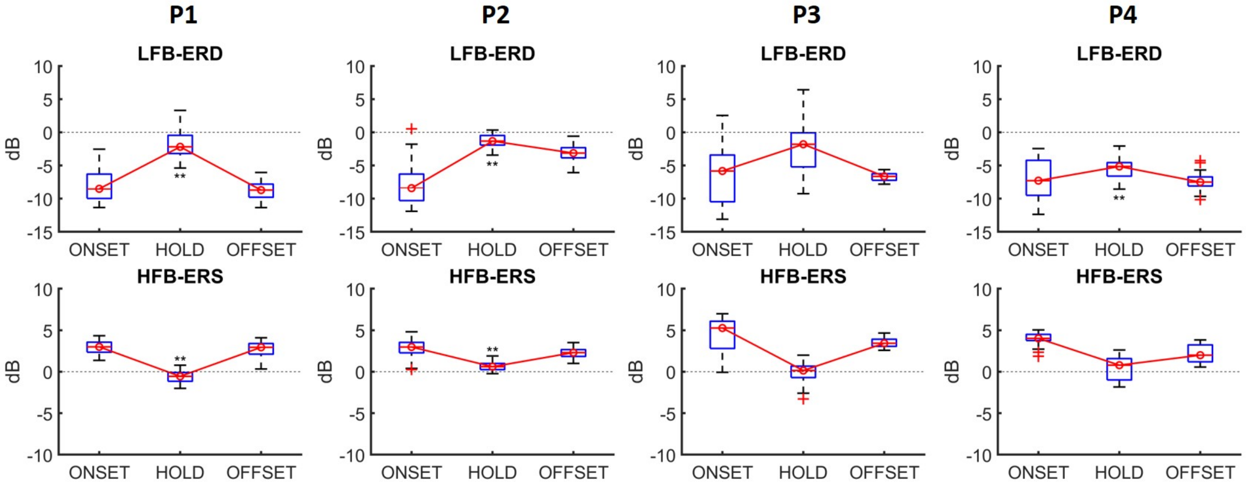

Figure S3: The boxplots show LFB-ERD and HFB-ERS magnitudes around grasp onset, hold, and offset phases for all four patients. The ERD plots are displayed from -15 to 10 dB and the ERS plots are displayed from -5 to 10 dB. Red crosses denote the outlier, red circles denote the mean values and the red horizontal lines denote the median values. The p-value of the test on whether LFB-ERD/HFB-ERS during hold is significantly different from baseline is provided. *: $p < 0.05$, **: $p < 0.01$.

Figure S3 shows the average LFB-ERD and HFB-ERS magnitudes around grasp onset, hold and offset phases. Two-tailed Student's t-test was used to determine whether the average LFB-ERD/HFB-ERS during the hold period was significantly different from baseline. Consistently in all patients, the magnitude of LFB-ERD has decreased and except P4, it almost returned to the baseline. The test results show that, except P3, LFB-ERD during hold was significantly smaller than baseline across all patients ($p < 0.01$), even though it had a much smaller magnitude than during grasp onset or offset. Similarly, during the hold phase, the magnitude of HFB-ERS has decreased consistently and returned to the baseline for P3 and P4. HFB-ERS was significantly higher than the baseline for P2 ($p < 0.01$), whereas it was significantly lower than the baseline for P1 ($p < 0.01$). Although P3 and P4 had slightly higher HFB-ERS levels than the baseline they were not significant (P3: $p = 0.07$, P4: $p = 0.058$).

3 CROSS-CORRELATION ANALYSIS OF ERD/ERS AND FORCE YANK FOR INDIVIDUAL PATIENT

Table S1 provides the mean and standard deviation of the cross-correlation values for the lag given in parenthesis across trials between HFB-ERS/LFB-ERD and force yank. The average lag for anterior (A) and posterior (P) channels of each patient at either grasp onset or offset is also provided in the table. During grasp onset, anterior and posterior LFB-ERD was negatively correlated with force yank, whereas HFB-ERD was positively correlated for all patients. The reverse pattern was true during the grasp offset. In addition, the lag between anterior/posterior LFB-ERD/HFB-ERS and force yank was positive during grasp onset for all patients. In contrast, during grasp offset the lag between anterior/posterior LFB-ERD/HFB-ERS and force yank was negative for all patients, except for the lag of anterior correlations for P2.

Table S1. Correlation and lag between LFB-ERD/HFB/ERS and force yank during grasp onset and offset and for each patient.

**ID**		**Grasp Onset**		**Grasp Offset**	
		Anterior	Posterior	Anterior	Posterior
LFB-ERD	P1	-0.62 ± 0.08 (17)	-0.62 ± 0.08 (15)	0.46 ± 0.11 (-141)	0.52 ± 0.10 (-120)
	P2	-0.63 ± 0.12 (50)	-0.61 ± 0.12 (6)	0.34 ± 0.24 (105)	0.31 ± 0.30 (-50)
	P3	-0.53 ± 0.11 (50)	-0.55 ± 0.16 (72)	0.37 ± 0.07 (-189)	0.33 ± 0.09 (-129)
	P4	-0.51 ± 0.13 (105)	-0.42 ± 0.19 (120)	0.25 ± 0.26 (-215)	0.21 ± 0.25 (-163)
HFB-ERS	P1	0.48 ± 0.13 (69)	0.79 ± 0.06 (29)	-0.50 ± 0.16 (-55)	-0.71 ± 0.10 (-93)
	P2	0.54 ± 0.16 (70)	0.75 ± 0.12 (60)	-0.11 ± 0.28 (60)	-0.43 ± 0.22 (-144)
	P3	0.68 ± 0.13 (151)	0.68 ± 0.18 (123)	-0.56 ± 0.10 (-120)	-0.57 ± 0.12 (-122)
	P4	0.74 ± 0.12 (142)	0.77 ± 0.10 (59)	-0.58 ± 0.12 (-122)	-0.60 ± 0.11 (-196)
